# Effects of CpG oligodeoxynucleotide 1826 on acute radiation-induced lung injury in mice

**DOI:** 10.1186/s40659-016-0068-5

**Published:** 2016-02-03

**Authors:** Xuan Li, Guoxiong Xu, Tiankui Qiao, Sujuan Yuan, Xibing Zhuang

**Affiliations:** Department of Radiation Oncology, Jinshan Hospital, Fudan University, Shanghai, China; Center Laboratory, Jinshan Hospital, Fudan University, Shanghai, China

**Keywords:** CpG ODN, Acute radiation-induced lung injury, Reactive oxygen species

## Abstract

**Background:**

The radiation-induced lung injury is a common complication from radiotherapy in lung cancer. CpG ODN is TLR9 activator with potential immune modulatory effects and sensitization of radiotherapy in lung cancer. This study aimed to examine the effect of CpG ODN on acute radiation-induced lung injury in mice.

**Methods and results:**

The mouse model of radiation-induced lung injury was established by a single dose of 20 Gy X-rays exposure to the left lung. The results showed that the pneumonia score was lower in RT+CpG group than in RT group on 15th and 30th days. Compared with RT group, CpG ODN reduced the serum concentrations of MDA (P < 0.05) and increased the serum concentrations of SOD, GSH (P < 0.05). The serum concentration of TNF-α in RT+CpG group was lower on 15th and 30th days post-irradiation (P < 0.05).

**Conclusion:**

The study demonstrated that CpG ODN has preventive effects of acute radiation-induced lung injury in mice. Lung inflammatory reaction and oxidative stress are promoted in the initiation of radiation-induced pneumonia. CpG ODN may reduce the injury of reactive oxygen species and adjust the serum TNF-α concentration in the mice after irradiation, which reduces the generation of the inflammatory cytokines.

## Background

Radiation therapy is one of the most important treatments for the chest tumors, but common complications from such treatments include radiation-induced lung injuries and dose-limiting side effects [1]. Cytosine–phosphate–guanine oligodeoxynucleotides (CpG ODNs) are synthetic DNA sequences containing unmethylated cytosine–guanine motifs, which are identified by activating Toll-like receptor 9 (TLR9) in antigen-presenting cells and B cells. CpG ODN can activate the active immune cells to produce a variety of cytokines which enhance the body’s specific and nonspecific immune effect and prevent a potential microbial infection [2]. It has been proven that CpG ODN can treat infectious diseases, cancer and allergic diseases [3], and also has good radiotherapy sensitization effect [4]. But there is no research on the effects of CpG ODN to acute radioactive lung injury. This study is a preliminary exploration on the effects of CpG ODN to acute radioactive lung injury in mice.

## Methods

### Experimental animals and reagents

One hundred and sixty ICR female mice were provided by the Shanghai Experimental Animal Center (Shanghai, China) and maintained in a specific pathogen-free grade animal room until 6–8 weeks of age and weighing 18–22 g. The study was approved by the ethics committee of Jinshan Hospital of Fudan University. CpG ODN1826 was purchased from Shanghai Biological Engineering Technology and Service Limited Company (Shanghai, China). CpG ODN1826 was completely phosphorothioate-modified and purified with PAGE gel. The sequence of CpG ODN1826 was: 5′-TCC ATG ACG TTC CTG ACG TT-3′. CpG ODN1826 was diluted in PBS to a concentration of 0.1 mg/ml. Mice serum malondialdehyde (MDA), superoxide dismutase (SOD), and glutathione (GSH) ELISA kits were purchased from Shanghai YAN JIN Scientific Research and Technology Limited Company (Shanghai, China). Mouse serum TNF-α ELISA kit was purchased from Millipore Company (Massachusetts, USA).

### Experimental groups

One hundred and twenty ICR mice were randomly divided into four groups: (1) Control group: intraperitoneal injection of 0.2 ml saline. (2) RT group: intraperitoneal injection of the same saline with single left lung 20 Gy of 6 MV X-ray. (3) CpG group: intraperitoneal injection of 0.1 mg/ml CpG ODN1826 solution 0.2 ml. (4) RT+CpG group: intraperitoneal injection of the same CpG ODN1826, with single left lung 20 Gy of 6 MV X-ray. The first injections were performed 12 h after radiation, which was defined as day 1. The saline or CpG ODN1826 was injected on days 1, 3, 5, 7 and 9, respectively. Six mice of each group were sacrificed on days 1, 5, 15 and 30.

### Radiation-induced lung injury mouse model

Before irradiation, mice were anesthetized by injecting intraperitoneal 4 μl/g body mass of 10 % chloral hydrate. The mice were immobilized and shielded under a home-made device. After the accurate positioning of irradiation area of mice with the simulator, single dose of 20 Gy of 6 MV X-rays was delivered to a 2 cm × 2 cm area in the left lung at a rate of 2.0 Gy/min. Non-irradiated mice underwent the same procedure but were not exposed to radiation.

### Pneumonia under optical microscope and estimating severity of pneumonia on a numerical scale

Mice were euthanized by an i.p. injection with an overdose of 10 % chloral hydrate. The lungs were fixated with 10 % formalin infusion through the tracheal cannula at a constant pressure of 25 cmH_2_O. After excision, the left lung was immersed in fresh fixative for at least 24 h, after which it was embedded in paraffin. After paraffin embedding, 5 μm sections were cut and stained with hematoxylin/eosin (H&E) according to standard methods. After H&E staining, observed the pneumonia under optical microscope. The Ashcroft scale was used for the quantitative histological analysis of pneumonia changes induced by irradiation. The severity of pneumonia was scored double blinded under optical microscope on the basis of Arrieta’s study [5], as follows: 0, normal; 1–2, alveolar walls slightly broadening, <10 inflammatory cells under high power field of vision; 3–4, alveolar walls significantly broadening, 10–20 inflammatory cells under high power field of vision; 5–6, alveolar walls significantly broadening, inflammatory cells infiltration full of alveolar walls; 7–8, in addition to the inflammatory cell infiltration full of alveolar walls, obvious alveolar infiltration. Each sample was scored with nine horizons randomly, and calculated the average score of each group finally.

### Detecting the serum MAD, SOD, GSH and TNF-α concentrations in mice

Mouse blood samples were collected under institute approved protocols and incubated at room temperature for 30 min to allow blood to clot. The blood samples were then centrifuged at 3000 rpm for 15 min. Sera were then collected and diluted 60-fold for testing MAD, SOD, GSH and TNF-α concentrations by ELISA.

### Statistical considerations

Statistical analysis was performed by SPSS21.0 (IBM). Data were expressed as means ± standard deviation (SD). Single factor analysis of variance test was used to test the differences between groups. The multiple comparisons were evaluated by the Bonferroni method. The non-parametric statistic test (Wald–Wolfowitz test) was used to test for differences between RT group and RT+CpG group on pneumonia scores. Differences resulting in *P* < 0.05 were considered to be statistically significant.

## Results

### Pneumonia under optical microscope

**Fig. 1:**
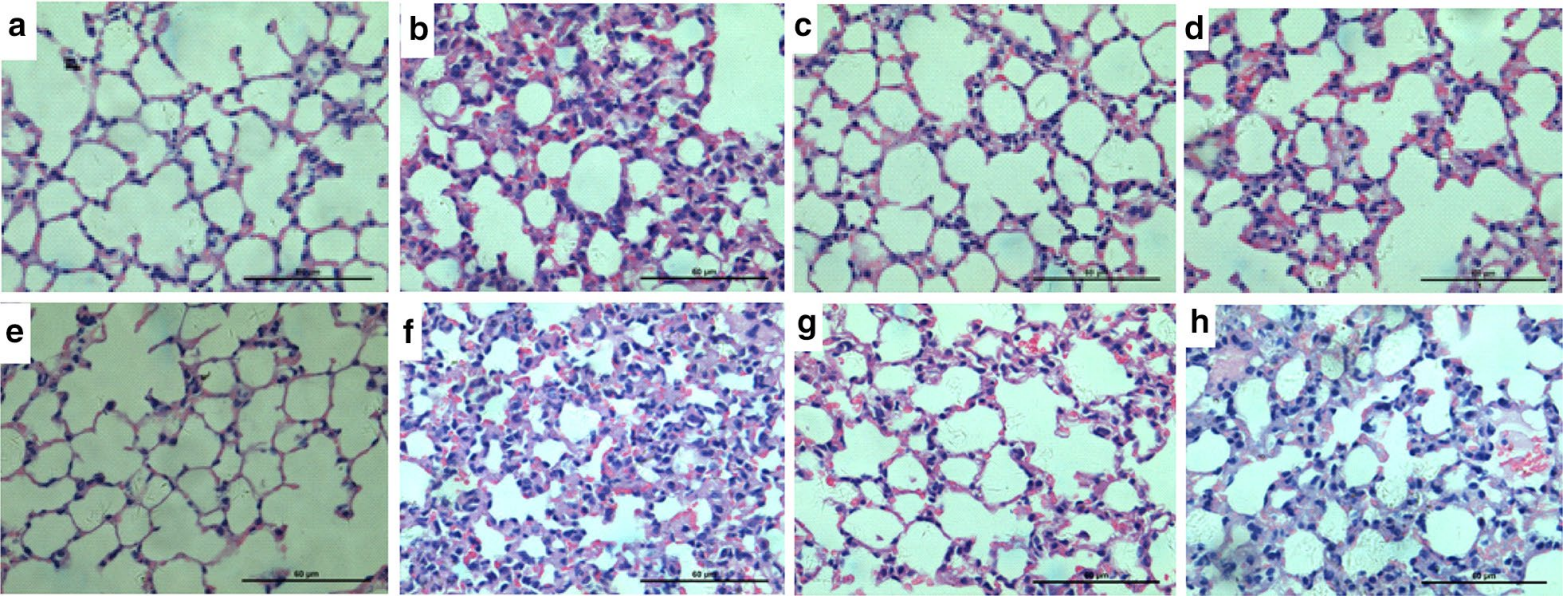
Left lung tissue paraffin section with HE staining (×400). **a** Control group days 15 after irradiation. **b** RT group days 15 after irradiation. **c** CpG group days 15 after irradiation. **d** RT+CpG group days 15 after irradiation; **e** Control group days 30 after irradiation. **f** RT group days 30 after irradiation. **g** CpG group days 30 after irradiation; **h** RT+CpG group days 30 after irradiation

**Fig. 2:**
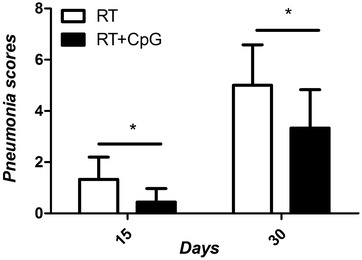
The scores of pneumonia on days 15 and 30. *P < 0.005, RT+CpG versus RT

Pleural effusion in the left chest was observed on days 30 in the RT and RT+CpG groups. There was no pleural effusion in pseudo irradiation groups. We observed the structural changes of bronchi, alveoli and alveolar interval organization and noted congestion, edema, inflammatory cell infiltration, and the degree of fibrosis under optical microscope Fig. (1). The HE staining of paraffin sections showed the bronchi, alveoli and alveolar interval structure of the pseudo irradiation groups were normal, with no chronic inflammation change. Obvious lung capillary hyperemia, mild alveolar septa broadening and edema, neutrophils and mononuclear macrophage infiltration of RT group were observed on days 15, and RT+CpG had lighter lung capillary hyperemia and less neutrophils and mononuclear macrophage infiltration than the other two irradiation groups on days 15. Part of the fiber cells and collagen fiber hyperplasia, a lot of neutrophils and mononuclear macrophage infiltration, and inflammatory cells gathered around secondary bronchi and blood vessels in the RT group on days 30. The RT+CpG group exhibited less pulmonary hemorrhage, the fiber cells and collagen fiber hyperplasia, neutrophils and mononuclear macrophage infiltration than RT group on days 30. The results in Fig. 2 showed the pneumonia score of RT group was higher than that of RT+CpG group on days 15 and 30.

### Effects of CpG ODN1826 to the serum MAD, SOD, GSH and TNF-α concentrations in mice

The levels of MDA are often used as an indication of oxidative damage and as a marker for free radical-induced for lipid oxidation, the more ROS, the higher levels of MDA. GSH and SOD, the primary defense, can reduce ROS [6, 7]. TNF-α is a key factor for tissue inflammation.

**Fig. 3:**
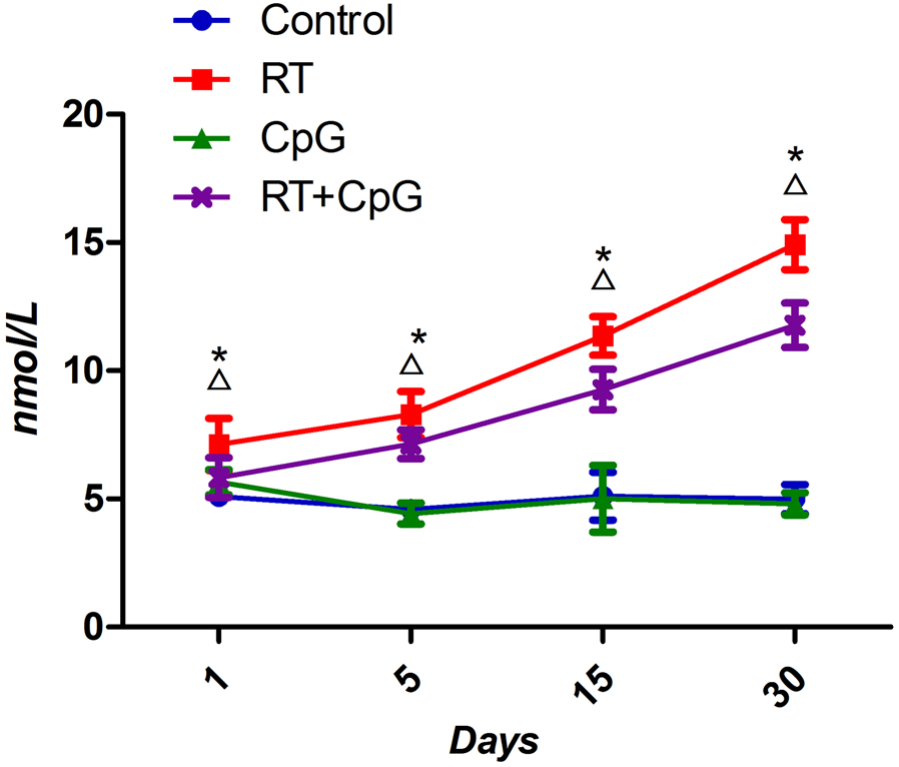
The serum MDA at different times. *P < 0.05, RT versus control; ^Δ^P < 0.05, RT+CpG versus RT

The results in Fig. 3 showed that serum concentration of MDA was higher in irradiation groups than that in pseudo irradiation groups and they kept rising after radiation, with the highest point on days 30. The serum concentration of MDA was lower in RT+CpG groups than that in RT group.

**Fig. 4:**
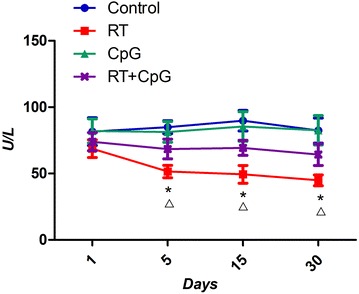
The serum SOD at different times. *P < 0.05, RT versus control; ^Δ^P < 0.05, RT+CpG versus RT

The results in Fig. 4 showed that serum SOD concentrations of irradiation groups were lower than that of the pseudo irradiation groups, and they kept decreasing after radiation, with the lowest point on days 30. The serum SOD concentrations of the RT+CpG groups were higher than that of RT group.

**Fig. 5:**
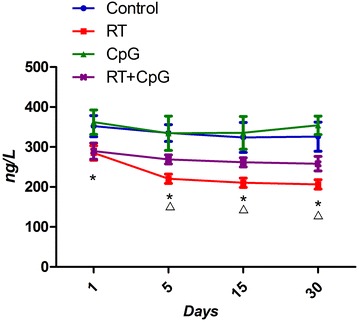
The serum GSH at different times. *P < 0.05, RT versus control; ^Δ^P < 0.05, RT+CpG versus RT

The results in Fig. 5 showed that serum GSH concentrations of irradiation groups were lower than that of the pseudo irradiation groups on, and they kept decreasing after radiation, with the lowest point was on days 30. The serum GSH concentrations of the RT+CpG groups were higher than that of RT group.

**Fig. 6:**
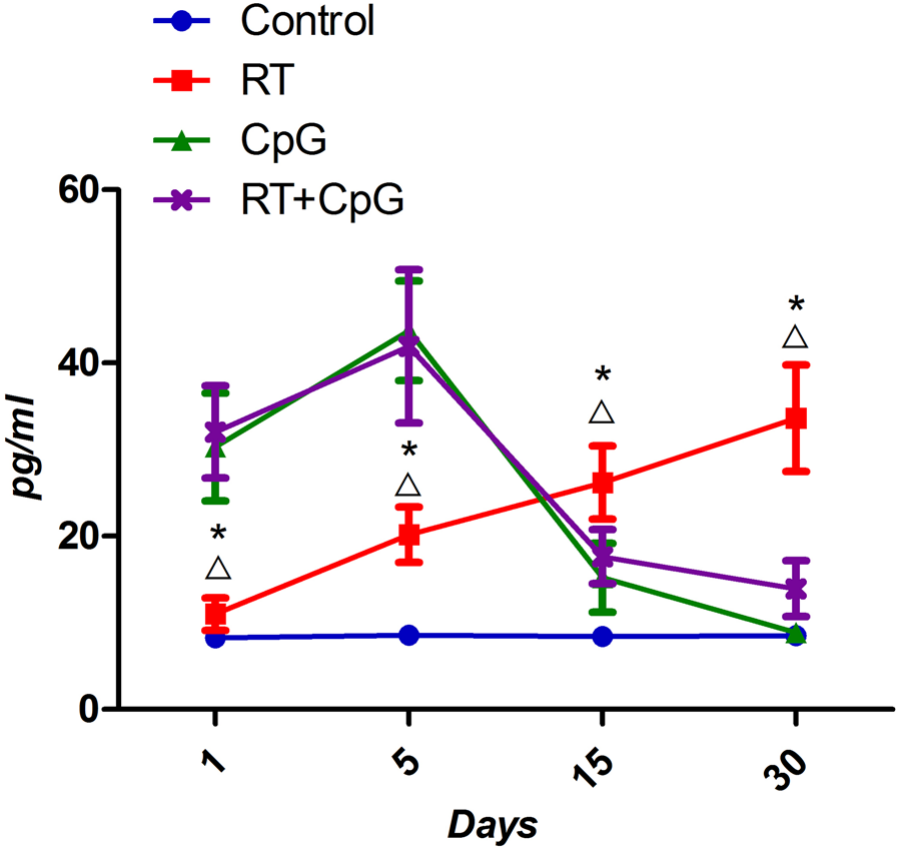
The serum TNF-α at different times. *P < 0.05, RT versus control; ^Δ^P < 0.05, RT+CpG versus RT

The results in Fig. 6 showed that the serum TNF-α concentrations of CpG and RT+CpG groups were higher than that of RT groups on days 1 and 5, but lower than that of RT group on days 15 and 30.

## Discussion

Radiation-induced pneumonia is the most common complication of radiotherapy in chest tumors. Increasing the radiation dose or the lung volume for treatment and combined with chemotherapy significantly increases the risk of radioactive lung injury, but its exact mechanism has not been fully elucidated. In the early-stage of damage, inflammatory mediator secretion increased continuously, causing alveolar exudates to increase, pulmonary interstitial hyperemia edema and inflammatory reaction cells infiltration. During the late stages of damage, tissue remodeling occurred and muscle fibroblasts increased, which resulted in collagen deposition and fibrosis [8, 9].

The role of Toll-like receptor (TLR) protein in immune-mediated responses is first discovered in drosophila in 1988 [10]. TLR9 is one of the important members of the TLRs family, and its activator is oligonucleotide with CpG as motif (CpG ODN). CpG ODN is synthetic DNA sequence containing unmethylated cytosine–guanine motifs that can imitate the bacteria or virus on stimulating the vertebrate immune system. CpG ODN now has a broad application prospect in the field of tumor treatment. It is not only used alone for tumor treatment, but also combined with a variety of other treatment methods, including immune therapy, radiation therapy and chemotherapy [11, 12]. It has been reported that CpG ODN has radio-sensitization effects. To our knowledge, there is no report on whether the CpG ODN participating in radiotherapy sensitization aggravates the acute radiation-induced lung injury. This study found that treatment of CpG ODN did not increase acute radiation-induced lung injury, but could treat the radioactive lung injury.

Recent studies suggest that the pathological morphological changes of radioactive lung injury mainly include pulmonary interstitial hyperemia and edema, an increase in alveolar exudates, inflammatory cells infiltration, late fiber connective tissue hyperplasia and alveolar atrophy [13, 14]. This study found that, after irradiation, the lung began to have congested capillaries, pulmonary edema, inflammatory cell infiltration late alveolar structure damage, and connective tissue hyperplasia and fibrosis. These changes in the RT+CpG group were not obvious. Therefore, CpG ODN improved the early symptoms of radioactive lung injury, increased lung tissue radiation resistance, and reduced pulmonary fibrosis at the same time to improve the prognosis.

Ionizing radiation can directly produce ROS and macrophages induced by actions such as the outbreak of breathing, indirectly generate ROS. ROS on the one hand directly attacks biological membrane of cells and tissues, increasing the alveolar capillary membrane permeability, causing leakage to increase between the lung tissue, and dissolving fibrin exudates. ROS also continues to stimulate fibroblast proliferation and collagen formation, leading to lung tissue degeneration and necrosis. On the other hand, ROS causes direct DNA damage mediated by intracellular signal transduction and gene expression in lung cell apoptosis, damage to biological macromolecules such as proteins, nucleic acids, lipids and reversible and irreversible damage to tissues and cells [15, 16]. The reduction in oxidative stress also plays a pivotal role in the radio-protective effect of CpG-ODN in radiated mice [17]. We know that radiation produces a harmful effect on living organisms by generation of ROS. The levels of MDA are often used as an indication of oxidative damage and as a marker for free radical-induced for lipid oxidation. The more ROS, the higher levels of MDA. GSH and SOD, the primary defense, can reduce ROS. Our results show that these ROS parameters (MDA, GSH, and SOD) demonstrated significant differences between the control group and RT group and RT+CpG group. CpG ODN could bind to TLR9 specifically, which activates the NF-kappa B pathway [18]. The activation of NF-kappa B can up-regulate proteins with antioxidant function, such as manganese superoxide dismutase (MnSOD) [19]. This may be a possible mechanism for CpG ODN to ameliorate the acute radiation injury of the lung.

TNF-α secreted by mononuclear cell and macrophage is an initiator for a variety of cytokines in regulating network, which play an important in the process of the occurrence and development of normal tissue inflammation [20, 21]. The study of Bao found that the alveolar macrophages, type II epithelial alveolar cells and bronchial epithelial cells of Sprague–Dawley rats exhibit strong TNF-α strong expression 2 h after irradiation, and this strong expression lasted for 4 weeks until it declined to normal levels [22]. CpG ODN is synthetic DNA sequence that can imitate the bacteria or virus on stimulating the vertebrate immune system. TNF-α is mainly secreted by Th1 cells, CpG ODN can stimulate Th1 to secrete TNF-α in the early stage, but when the concentration of TNF-α is too high, it can stimulate CpG ODN to inhibit the secretion of Th1, and to promote the secretion of Th2,CpG ODN can maintain the balance of Th1/Th2 [23, 24]. Thus, TNF-α concentrations of RT groups and CpG+RT groups both increased on days 1and 5, but TNF-α concentrations of RT+CpG groups declined rapidly on days 15 and 30, and TNF-α concentrations of RT groups still increased. Therefore, CpG ODN can adjust the serum TNF-α concentration in the mice after irradiation, which reduces the generation of the inflammatory cytokines, and ameliorate the radiation injury of the lung.

Since ionizing radiation can directly produce ROS, and ROS directly attacks biological membrane of cells and tissues. ROS is the direct cause of radiation-induced pneumonia [25]. In addition, the cell damage caused by direct radiation can also initiate the cascade reaction of inflammatory cytokines, and induce the continuous generation of endogenous ROS, and the effect of TNF-α is the strongest [26]. Our study also found that MDA, GSH (makers of ROS) and TNF-α concentrations of irradiation groups kept rising after radiation, with the highest point on days 30. Thus, lung inflammatory reaction and oxidative stress are promoted in the initiation of radiation-induced pneumonia.

## Conclusions

In conclusion, our study demonstrated that CpG ODN has preventive effects of acute radiation-induced lung injury in mice. Lung inflammatory reaction and oxidative stress are promoted in the initiation of radiation-induced pneumonia. CpG ODN may reduce the injury of reactive oxygen species and adjust the serum TNF-α concentration in the mice after irradiation, which reduces the generation of the inflammatory cytokines.

## Abbreviations

CpG ODN1826: CpG oligodeoxynucleotide 1826
MDA: malondialdehyde
SOD: superoxide dismutase
GSH: glutathione
ROS: reactive oxygen species
TNF-α: tumor necrosis factor-α
